# The effect of match location on hydration status in Division I women’s soccer athletes

**DOI:** 10.1080/15502783.2025.2550156

**Published:** 2025-09-02

**Authors:** Toni L Brem, Savannah J Noll, Patrick G Saracino

**Affiliations:** The University of South Carolina Upstate, Department of Human Performance and Health, Spartanburg, SC, USA

**Keywords:** Collegiate soccer, competition, hydration, travel

## Abstract

**Background:**

Athletes must frequently travel for away games during their competitive season. Previous work suggests that travel adversely impacts multiple factors relevant to optimal athletic performance. However, no research has examined the influence of match location on the hydration status of Division I women’s soccer athletes. Therefore, the purpose of this study was to assess hydration status in a Division I women’s soccer team during home and away matches.

**Methods:**

Urine samples were collected ≤2 h prior to one away game and two home games during the 2025 Spring season (Mar–April). Then, wearing compression shorts and sports bras, athletes were asked to remove excess sweat using a towel prior to performing a body weight (BW) measurement via a portable bioelectrical impedance analysis scale (InBody H30). Urine samples were analyzed immediately following BW for urine color (UC) and urine specific gravity (USG) using a handheld refractometer (Atago, PAL-ATHLETE). USG values ≥1.020 were considered hypohydrated. UC was compared to validated UC charts with samples held against a white background in a well-lit room. BW was measured again within 30 min following the match. Dehydration, as a percentage of BW, was calculated as: $\mathrm{Dehydration}\left(\%\mathrm{BW}\right)=\left[\frac{(\mathrm{Pre}-\mathrm{eventBW}\left(\mathrm{kg}\right)-P\mathrm{ost}-e\mathrm{ventBW}\left(\mathrm{kg}\right)}{\mathrm{Post}-\mathrm{eventBW}\left(\mathrm{kg}\right)}\right]\times 100$

**Results:**

Eleven field players (forward, midfield, defender) participated in this study (166.1 ± 5.9 cm, 61.0 ± 5.8 kg, 23.8 ± 4.0% body fat, 25.8 ± 2.6 kg SMM). Environmental conditions for home and away matches were 31.6°C, 30% RH and 23.3°C, 37% RH, respectively. Combined pre-competition USG and UC were 1.009 ± 0.007 and 2.3 ± 1.4, respectively. Athletes lost 2.3 ± 0.8% BW during competition. Hypohydration prevalence was 5% and 18% prior to home and away games, respectively. USG was significantly lower at home (1.008 ± 0.007) compared to away (1.012 ± 0.005) matches (*p* = 0.032). There were no differences for UC (2.1 ± 1.6 vs. 2.6 ± 0.9) or dehydration (2.4 ± 0.9 vs. 2.1 ± 0.7% BW) by match location.

**Conclusions:**

In a Division I Women’s Soccer team participating in sports nutrition education, most athletes begin competition in an appropriately hydrated state. Though differences in USG were observed, mean values for home and away matches were considered hydrated. Accordingly, road travel <3 hours does not alter indices of pre-competition hydration status or dehydration in Division I women’s soccer athletes.

